# Optimization of Joint Decision of Transport Mode and Path in Multi-Mode Freight Transportation Network

**DOI:** 10.3390/s22134887

**Published:** 2022-06-28

**Authors:** Yang Lu, Shuaiqi Wang

**Affiliations:** 1 Business School, Hohai University, Nanjing 211000, China; 2 School of Transportation, Southeast University, Nanjing 211189, China; siqiwangseu@163.com

**Keywords:** multi-mode transportation, joint decision, optimization, network, freight

## Abstract

This paper mainly studies the joint decision of transportation mode and path in the multi-mode transportation network to provide the optimal plan for freights. This paper constructs a multi-mode transportation network system by setting virtual connections between networks with different transportation modes. The Dijkstra and multi-objective optimization algorithms are used to select the path in the network. After determining the optimal path, the paths’ time, cost, and risk functions are established. The multi-objective function is converted into a single objective function by setting constraint conditions through the analytic hierarchy process. Then, the function is optimized by using the gradient descent method. Finally, the transportation plan for the case of chemical freights is formulated by using the above algorithms. The results show that the proposed algorithm can successfully find the solution for the joint decision of transportation mode and path in the complex network. After a quantitative analysis of the planned effect, the optimization actions of changing the initial transportation time and adjusting the upper limit of resources are proposed. The study findings provide a theoretical basis for improving the efficiency of the comprehensive transportation network.

## 1. Introduction

Multi-mode freight transportation has become a hot topic for researchers worldwide in modern society [1,2,3]. It is also necessary for the sustainable development of society and the economy in various countries. In the analysis, the key to the joint decision of transportation mode and path in multi-mode transportation networks is to find an efficient plan in the comprehensive transportation system [4,5]. There are usually five modes: railway transportation, road transportation, sea transportation, air transportation, and pipeline transportation. The joint decision can fully play the advantages of different transportation modes, providing better services, achieving a convenient circulation of goods, and increasing transportation service providers’ economic value [6,7].

Many theoretical studies on multi-mode transportation do not consider joint decision modeling, especially for medium and long-distance multi-mode freight transportation. Due to the complex process, path selection and plan formulation have several challenges: (1) The problem of transportation mode and path selection in a multi-mode transportation network is a dynamic random process in which complex logistics, information flow, and capital flow are intertwined [8]. Studying the influencing factors of decision making and proposing the evaluation approach of the transportation plan can help promote the rational development of multi-mode transportation. (2) The path design of multi-mode transportation is related to the efficiency of economic growth and the full utilization of transportation resources, which requires careful consideration of multiple objectives [9].

The significance of this paper is to develop an optimization approach for making freight transportation plans for multi-mode transportation networks. The joint decision and evaluation technology of transportation mode and path are proposed. First, a multi-mode transportation network system is constructed by setting virtual connections between networks with different transportation modes. The Dijkstra and multi-objective optimization algorithms are used for path selection in this network. After determining the optimal path, the path’s transportation time, cost, and risk functions are established. The multi-objective function is converted into a single objective function by setting constraint conditions through the analytic hierarchy process. Then, the gradient descent method is used to optimize the procedure. Finally, the overall transportation plan for the case of chemical transportation is formulated using the above algorithm, and the plan’s effect is quantitatively analyzed. The optimal plan can shorten transportation time and reduce transportation costs to improve the benefit of the whole transportation network.

## 2. Literature Review

Among the analysis of the multi-mode transportation network, the influencing factors of path selection are the primary condition to ensure the rationalization of transportation. We can set a proper objective function to meet transportation service providers’ and demanders’ economic needs by selecting relevant factors. In this regard, many scholars have carried out the research.

After describing the multi-mode transportation network, Zhang et al. [10] established the optimal allocation model of multi-mode transportation with transportation cost as the influencing factor, quantitatively analyzed the transportation organization plan, and explained the impact of transportation cost on transportation mode and transportation path. In the multi-mode path selection, Wei et al. [11] considered the influencing factor of transportation cost, mainly including the transit cost between different transportation modes. They also considered the influencing factor of transportation time, mainly including the transit time of goods between different transportation modes. They put forward the shortest path model under real-time change and made a solution.

Su et al. [12] regarded transportation time, transportation cost, and quality as the key factors influencing multi-mode transportation. They mainly put forward the impact of transportation quality on plan formulation and used the analytic hierarchy process to add quantitative calculations based on qualitative analysis. Considering the main factors such as cargo accident probability and accident consequences, Wei et al. [13] studied the optimization design of freight transportation paths. They introduced the risk model in the formulation. Compared with the traditional shortest path model, its plan can effectively reduce the risk and provide a new idea for analyzing influencing factors of path selection.

In the multi-mode transportation path decision-making method, Yang et al. [14] used the nonlinear bilevel programming theory to plan the transportation network and constructed a path optimization model considering transportation time and cost. Lozano and Storchi [15] and others used the label method to prepare the shortest path, used the modified continuous time series method to solve the shortest possible path in multi-mode transportation, and set certain constraints to filter the practical and feasible path.

The risk influencing factors of goods transportation is also significant. Due to the different physical environments of roads and sections, the probability of accidents is also distinct [16,17]. For the weight analysis of various influencing factors, Li and Liu [18] used an analytic hierarchy process to determine the weights according to the multi-layer attributes of transportation risk, cost, and time of freights in road transport [19,20]. Zhang [21] constructed a multi-modal dangerous goods transport planning model targeting transport costs and transport risks to achieve low-cost and low-risk delivery of goods. Hartlage [22] designed a new transport capacity allocation method based on the resource-constrained shortest path problem (RCSP) according to the characteristics of the transport network, delivery schedules, transport costs, and other conditions. Yang et al. [23] constructed a cargo transport capacity model for a multi-modal multi-path transport network with the maximum cargo capacity as the objective. They designed a genetic algorithm incorporating an adaptive process to solve the model according to the characteristics of the constructed model. Boussedjra et al. [24] used a two-way study approach to consider the multi-modal multi-path transport network problem. They created a shortest path optimization model to minimize the time required to transport the goods.

Garrido and Bronfman [25] studied route planning for the road transport of dangerous goods of various categories in urban areas, focusing on the balance of risk impacts and social acceptance in the areas they passed through, taking into account factors such as the occurrence of accidents, the balanced distribution of the affected population, regular transport cycles, risk permit levels, and differences in risk distribution. Fan et al. [26] studied the route planning and vehicle scheduling of dangerous goods road transport in urban areas, taking into account the influence of objective conditions such as traffic congestion and traffic control at the city-region scale, whose risk measurement factors include the probability of accidents and the number of the exposed population, and further developed a bi-objective mixed-integer nonlinear programming model.

Other independent factors that affect the transport processes and cannot always be directly included in the model could also be considered. Those factors could be the kinematics of vehicle movements [27,28] and local infrastructure that affects the ability to move vehicles [29,30,31], which affects vehicle driving safety [32], as well as the time of road transport services. For example, overcoming obstacles on the road [33] and the impact of the suspension or the strategy of making decisions [34,35] are highly related to the risk of a reduced coefficient of adhesion [36,37], e.g., under snow-covered road conditions. In addition, there are psychophysical conditions of the driver, such as fatigue, stress, or random events [38]. Those factors may have a random disturbance affecting the adopted variant, e.g., the route, and are recommended to be considered in the modeling if data are available.

Though previous studies have proposed several models for transportation decision optimization, they do not fully consider the joint decision of transportation mode and path. The transportation of goods will inevitably cause traffic loss, including capital loss and time loss. In addition, no transportation plan fully considers the transportation risk, cost, and time in the joint decision of a multi-mode freight plan. Previous studies do not seek the multi-objective integration methods to achieve the optimal effect.

## 3. Methodology

### 3.1. Construction of Multi-Mode Transportation Network

**Figure 1:**
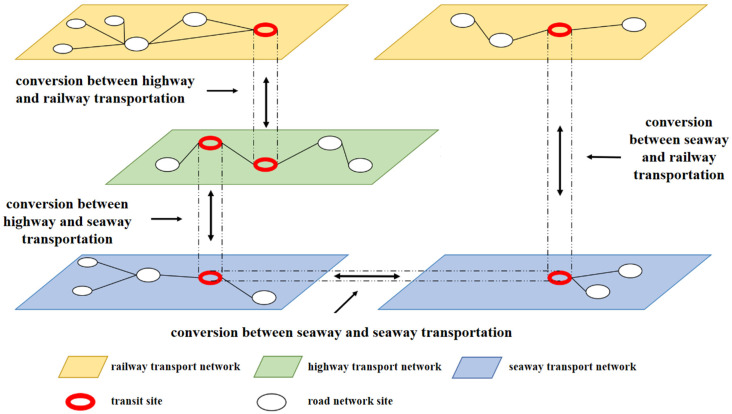
Structure diagram of integrated transportation network.

The network in multi-mode transportation mainly includes a road transportation network, railway transportation network, waterway transportation network, and civil aviation transportation network. Civil aviation transportation is point-to-point, and the path selection space is minimal. Therefore, it is not considered in multi-mode transportation modeling in our current study. This paper mainly aims to construct a network system for the three modes of transportation. The structure diagram of the integrated transportation network is shown in Figure 1.

The three transportation networks are divided into three layers for combination modeling in this system. Each layer includes high-quality stations and paths, represented by the real connection, road number, road network length, limited speed and passing capacity, and a specific attribute value. Virtual links are set between layers to represent the cost of replacing the two transportation modes, while specific attribute values should be given.

**Figure 2:**
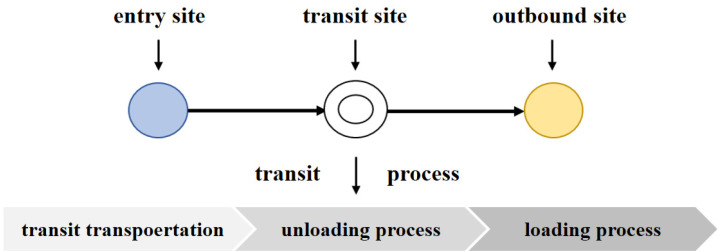
Workflow of integrated transportation network.

In the calculation process, to make the calculation more convenient, the three-layer comprehensive transportation network system needs to be compressed into a one-layer plane system. Therefore, it is necessary to add a transportation node based on the original transportation network and set the transshipment process and transportation process as a unified order of magnitude to represent the whole transportation process. The specific transportation network units are shown in Figure 2.

In this transportation unit, goods enter from the entry site to the transit link, and the transfer site is treated as a transportation node. By transforming the transfer time, loading and unloading equipment cost, loading and unloading personnel cost, and other attributes of the transfer site into the attributes between road sites, the plane synthesis of a single weight can be realized. The transit process can be divided into three stages: transit transportation, unloading, and loading.

### 3.2. Dijkstra Algorithm for the Shortest Path Problem

**Figure 3:**
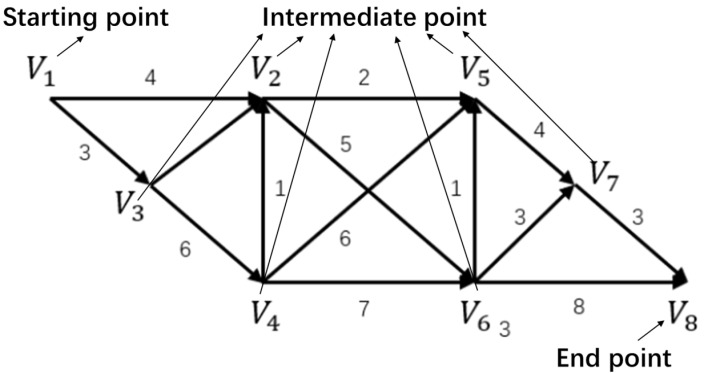
Transportation network diagram including nodes and links.

The so-called shortest path problem is to find a path with the shortest line length from a starting point to an ending point in a network, where the line length between adjacent nodes is known. An example is the following network diagram (see Figure 3) to find the shortest path between the starting point V1 and the endpoint V8.

This paper mainly uses an algorithm proposed by Dijkstra [39,40], which is recognized as a good algorithm for our modeling purpose. This algorithm is called the labeling method or the Dijkstra algorithm. It can find the shortest path from the starting point V1 to the end point V8 and obtain the shortest path from the starting point to any point.

First, from the starting point V1, we mark a number for each vertex called label; they are divided into T label and P label. The T label represents the upper bound of the shortest path weight from the starting point to this point, called the temporary label. The P label represents the shortest path weight from the starting point to this point, which becomes a fixed label. The points labeled P will not change, and those not labeled P will be labeled T. Every algorithm step will change the T label to the P label. After small steps, all points can be labeled P, and the shortest path weight from the starting point to each point can be obtained.

The specific calculation steps are as follows:

Step 1: Label V1 with P, P (1) = 0, and label other points with T. T0 (j) = +∞ indicates from V1 to V1, the shortest path weight is 0, and the upper bound of the shortest path weight from V1 to each point is +∞. The number in parentheses represents the point number in the label, and the corner code 0 illustrates the initial value.

Step 2: Assuming Vi as the previous round label (the k − 1 round label) has just obtained the P label, then a new round of labeling is performed for all points that have not received the P label (round K). Considering all points Vi that are adjacent to Vj and not labeled P, we modify the T label:

Tk(j) = min [T(j), P(i) + dij](1)

where:

dij—distance (weight) from Vi to Vj;
T(j)—the T label obtained at the point Vj before the k-round labeling.

Among all T labels, we find the smallest T label T(j) as follows:

Tk(j0) = min[Tk(j),Tl](2)

where:

Tl—V1 nonadjacent points Vi obtained with T number.

We then mark the point Tj0 with P label:

P(j0) = T(j0)(3)

Step 3: If there is no T label point in D, the algorithm ends. Otherwise, proceed to step 2.

### 3.3. Multi-Objective Optimization Algorithm for Solving Optimal Path

Path optimization is considered a multi-objective optimization problem, and the optimization result is affected by several factors such as transportation cost, transportation time, and transportation risk. The optimization of the plan concerning the derived single-objective problem is expected to realize a win–win situation among transportation managers, operators, and consumers and promote the development of the comprehensive transportation industry. In such a way, the transportation resources and human labor can be utilized in a balanced way. Due to the mutual restriction and conflict between various factors, it cannot be guaranteed that when the target value of one element is optimal, the target value of other factors can also meet the conditions of optimization. Thus, there is no absolute optimal solution for this optimization goal. Therefore, the solution of the model is mainly to find a solution with the optimal result that considers the comprehensive factors so that the single objective function transformed from the multi-objective function is optimal. The specific algorithm steps are as follows:

Step 1: Build a multi-mode transportation network. First, find a feasible path from the transportation starting point to the endpoint based on the open-source map. Then, label the starting point, the endpoint, and transit point, divide the transportation network into V stages, and mark each stage’s path length and transportation mode.

Step 2: On this network, calculate the optimal path with the shortest transportation distance, the minimum transportation risk, and the shortest transportation time as the single objective. If the three paths are the same, determine that the path is optimal and end the algorithm; otherwise, go to step 3.

Step 3: Calculate the total index value (which includes distance, risk, and time) of each road section and select the optimal path.

Step 3.1: Write the index value matrix of all road sections 𝐴 = (𝑎ij) 𝑚 × 𝑛, where m and n are the number of start site i and end site j, respectively. Among them, to eliminate the influence of dimension and make different variables comparable, the index value matrix should be normalized to 𝑅 = (rij) 𝑚 × 𝑛, and the processing method is r_ij = (a_max − a_ij)/(a_max − a_min).

Step 3.2: Obtain the total index values (in terms of transportation distance, risk, and time) of different paths according to different weights assigned to indexes, which decide the relative importance of transportation distance, risk, and time. Select the path of the smallest total index value as the optimal path.

## 4. Experimental Design

### 4.1. Transportation Plan Model

#### 4.1.1. Model Assumptions

It is assumed that a transporter needs to transport one or more batches of goods from city A to city B within a period, passing through n cities on the way. Different transportation modes can be selected in other sections, and the costs, risks, and time incurred when using different transportation modes in other areas are different. The specific assumptions are as follows:

- Only one mode of transportation can be selected between two transfer stations.
- Goods can only be transported in batches without disassembly and assembly between nodes.
- There are resource constraints such as transportation time, transportation cost, maximum risk, personnel, and equipment in the whole transportation process.
- Considering the changes in various indicators under time-varying conditions (which means parameter values can be different in a different time), plans are made under typical scenarios.
- The maintenance of personnel and equipment between two means of transport is not considered.
- The number and characteristics of vehicles are not considered.
- Because the loading and unloading risk is much smaller than the transportation risk, the loading and unloading risk will not be considered when calculating the total transportation risk.
- It is assumed that the goods will be loaded and unloaded immediately after arriving at the transfer site. The following transportation section is carried out directly after loading and unloading, without intermediate waiting time.

#### 4.1.2. Notation

The overall transportation volume is M (the unit is t), the transportation distance of each section is Ln (the unit is km), the number of people transported in each section is Fn, and the number of leased loading and unloading equipment at each node is Sn. G (N, K) is a transportation network containing multiple transportation paths and modes; N is the set of transportation network nodes, n ∈ N; K is the set of all transportation modes; (K1, K2) ∈ K represents the transfer between the two transportation modes; C_(n,n + 1)^k^ is the transportation cost of selecting transportation mode k between sites n and n + 1 for the transportation goods (the unit is yuan), C(n,n + 1,K) is the fixed transportation cost of selecting transportation mode k between sites n and n + 1 for the transportation goods (the unit is yuan·((km·t)^−1^); Q_n^(k_1,k_2)^ is the transfer fixed cost of goods from transportation mode k1 to transportation mode k2 at the site n (the unit is yuan). For loading and unloading cost: En is the cost of goods using loading and unloading equipment at site n (the unit is yuan·(set·h)^−1^), and (Pn,n + 1) is the unit labor cost of personnel transporting goods between sites n and n + 1 (and the unit is yuan (people·h)^−1^).

T_(n,n + 1)^k^ is the unit transportation time of selecting transportation mode k between sites n and n + 1 for the transportation of goods (the unit is h·km^−1^); T(n, k1, k2) is the unit transit time of goods at site n (the unit is h·t^−1^); R_(n,n + 1)^(k,i)^ is the scoring value of the i-th influencing factor when the goods are transported by selecting the transportation mode k between sites n and n + 1; ri is the weight of the i-th risk influencing factor (i = 1,2,3,4). Tl is the maximum limited transportation time, the unit is h; Cl,t is the upper budget limit of the whole transportation process, the unit is yuan; Cl,e is the upper budget limit of the entire loading and unloading process; Rl is the upper limit of risk tolerance for the entire transportation process. The maximum number of transportation personnel is F1, and the maximum loading and unloading equipment is Sl. The following equations are used to define *x* and *y*, respectively:

(4) $$x_{n,n+1}^{k}=\left\{\begin{array}{c} 1\;\;\;select\;transportation\;mode\;k\;between\;sites\;n\;and\;n+1\\ 0\;\;\;others \end{array}\right.$$

(5) $$y_{k1,\;k2}^{n}=\left\{\begin{array}{c} 1\;\;\;two\;transit\;modes\;k1\;and\;k2\;at\;the\;site\;n,\\ 0\;\;\;others \end{array}\right.$$

#### 4.1.3. Model Building

Because the cost, time, and risk of each stage are unstable, it is only necessary to quantify the indicators of each stage and sum them up. The specific model is as follows: 

Cost model function:

(6) $$\begin{array}{c} C=\sum_{k_{1}}\sum_{k_{2}}\sum_{n}y_{k_{1},k_{2}}^{n}\cdot Q_{n}^{k_{1},k_{2}}+\\ \sum_{k}\sum_{n}x_{n,n+1}^{k}\left[C_{n,n+1}^{k}+C\left(n,n+1,k\right)\cdot L_{n}\cdot M\cdot +E_{n}\cdot S_{n}\cdot T\left(n,k\right)+P_{n,n+1}\cdot T_{n,n+1}^{k}\cdot F_{n}\right] \end{array}$$

Time model function:

(7) $$T=\sum_{k}\sum_{n}x_{n,n+1}^{k}\cdot T_{n,n+1}^{k}\cdot L_{n}+y_{k_{1},k_{2}}^{k}\cdot T\left(n,k_{1},k_{2}\right)\cdot M$$

Risk model function:

(8) $$T=\sum_{k}\sum_{n}x_{n,n+1}^{k}\cdot \left[r_{1}\cdot R_{n,n+1}^{k,1}+r_{2}\cdot R_{n,n+1}^{k,2}+r_{3}\cdot R_{n,n+1}^{k,3}+r_{4}\cdot R_{n,n+1}^{k,4}\right]$$

Objective function:

(9) $$\mathrm{Min}\;Z=\alpha_{1}\cdot C+\alpha_{2}\cdot T+\left(1-\alpha_{1}-\alpha_{2}\right)\cdot R$$

Constraints:

(10) $$\sum_{k}x_{n,n+1}^{k}=1,\forall k\in K,\forall n\in N$$

(11) $$\sum_{k_{1}}\sum_{k_{2}}y_{k_{1},k_{2}}^{n}=1,\forall k_{1},k_{2}\in K,\forall n\in N$$

(12) $$\sum_{k_{1}}\sum_{k_{2}}y_{k_{1},k_{2}}^{n}\cdot Q_{n}^{k_{1},k_{2}}\leq C_{l,e},\forall k_{1},k_{2}\in K,\forall n\in N$$

(13) $$\begin{array}{c} \sum_{k}\sum_{n}x_{n,n+1}^{k}\left[C_{n,n+1}^{k}+C\left(n,n+1,k\right)\cdot L_{n}\cdot M+E_{n}\cdot S_{n}\cdot T\left(n,k\right)+P_{n,n+1}\cdot T_{n,n+1}^{k}\cdot F_{n}\right]\leq C_{l,t},\\ \forall k\in K,\forall n\in N \end{array}$$

(14) $$\sum_{k}\sum_{n}x_{n,n+1}^{k}\cdot T_{n,n+1}^{k}\cdot L_{n}+y_{k_{1},k_{2}}^{k}\cdot T\left(n,k_{1},k_{2}\right)\cdot M\leq T_{l},\forall k\in K,\forall k_{1},k_{2}\in K,\forall n\in N$$

(15) $$\sum_{k}\sum_{n}x_{n,n+1}^{k}\cdot \left[r_{1}\cdot R_{n,n+1}^{k,1}+r_{2}\cdot R_{n,n+1}^{k,2}+r_{3}\cdot R_{n,n+1}^{k,3}+r_{4}\cdot R_{n,n+1}^{k,4}\right]\leq R_{l}$$

(16) $$\sum_{n}F_{n}\leq F_{1},\forall n\in N$$

(17) $$\sum_{n}S_{n}\leq S_{1},\forall n\in N$$

(18) $$x_{n,n+1}^{k}\in \left\{0,1\right\},\;y_{k_{1},k_{2}}^{n}\in \left\{0,1\right\},\forall k\in K,\forall k_{1},k_{2}\in K,\forall n\in N$$

(19) $$M\geq 0,L_{n}\geq 0,F_{n}\geq 0,S_{n}\geq 0,\forall n\in N$$

In the above model, Formula (6) represents the cost model function, including transfer cost, fixing cost, variable cost, equipment rental cost, and personnel labor cost. Formula (7) represents the time model function, including transportation time and transfer time; Formula (8) represents the risk model function, which is the weighted sum of the four indicators of accident probability, population density, road impedance, and emergency rescue capability in the path; Formula (9) is the objective function of path selection, and the weight is introduced according to the importance of transportation cost, transportation time, and transportation risk to convert the multi-objective function into a single objective function; Formula (10) is the selection constraint on the path, and there can only be one path between two network nodes t; Formula (11) is the constraint on the choice of mode of transportation, and there is only one transportation mode on a section of the path; Formula (12) indicates that the cost of the whole loading and unloading process shall not exceed the loading and unloading upper limit, because the number of transportation equipment is restricted; Formula (13) means that the cost of the whole transportation process shall not exceed the upper cost limit to ensure a particular economy; Formula (14) means that the time of the entire transportation process shall not exceed the upper time limit to ensure specific efficiency; Formula (15) means that the risk of the whole transportation process shall not exceed the upper risk limit to meet the social requirements; Formula (16) indicates the boundary of the number of personnel used in the whole transportation process; Formula (17) shows the limit of the number of loading and unloading equipment used in the entire transportation process; Formula (18) is the constraint of decision variables; Formula (19) is the nonnegative constraint of variables.

Note that the correlation related to the objective factors is essential to the modeling structure. Our study calculates the risk, time, and distance values for all candidate paths with different environment variables and conducts the Pearson correlation tests. The Pearson’s correlation coefficient, which is the covariance of the two variables divided by the product of their standard deviations, among risk, time, and distance for candidate paths, is 0.13, 0.21, and 0.18, respectively, indicating that these factors are not closely correlated. The reason is that the values of the above three factors are a combination of the characteristics of each transportation mode, the features of each transportation route, the attributes of each transportation tool, and the loading and unloading of each transit node, rather than the simple linear correlation between them. Thus, the single-objective optimization model is considered in our research.

Dijkstra’s algorithm is a method of the shortest path in graphs, especially for weighted graphs. It is only suitable for finding the shortest path with positive edge weights. Generally, it is a single-source-directed weighted graph. This shortest path algorithm goes from one vertex to the other vertices, which solves the shortest path problem in the weighted graph. The main feature of Dijkstra’s algorithm is that it starts from the starting point and adopts the strategy of a greedy algorithm.

The shortest path in the text does not simply mean the shortest physical distance between two points; it is defined as the path with the most excellent utility. It considers a combination of financial costs, execution times, transport risks, etc., which are presented in the calculation function in Section 4.1.3. In addition to this, it can also be calculated using Floyd’s algorithm. As with Dijkstra’s algorithm, Floyd’s algorithm aims to find the shortest path between vertices in a given weighted graph. In contrast to Floyd’s algorithm, Dijkstra’s algorithm is not able to handle graphs with negatively weighted edges and negatively weighted loops, but Dijkstra’s algorithm has good scalability and can be adapted to many problems, especially those with large amounts of data, and is the most commonly used shortest path algorithm.

## 5. Analysis of Optimization of Transportation Plan in Multi-Mode

### 5.1. Results of Case Study

**Figure 4:**
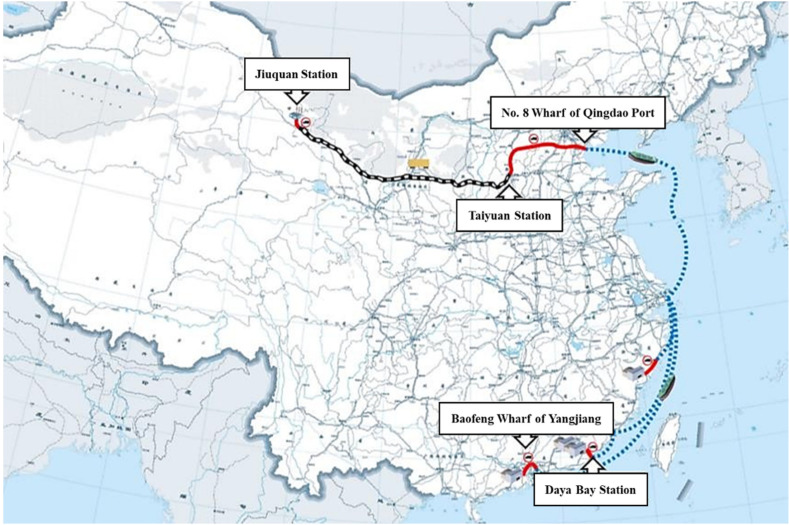
A numerical example of the joint path selection plan for transportation.

A numerical example is shown in Figure 4. The transportation of this case starts from Daya Bay station on the southeast coast and ends at Taiyuan station in the northwest. There are numerous candidate routes for different transportation models (they are not shown in the figure, due to the complexity). During the journey, it has experienced at least two modes of transportation: road transportation and railway transportation, and, at most, three modes of transportation: road transportation, railway transportation, and sea transportation.

According to the initial route screening method mentioned above, there are two paths after the first screening: the first is pure inland transportation without sea transportation, and the second is a mixed-path plan of sea transportation, road transportation, and railway transportation. In our optimal function, we need to reach the global optimization among different factors. For example, we found in this example that the path of pure inland transportation is shortest. Still, the transportation cost is substantial and does not meet the global optimization requirement. An example of the joint path and mode selection result is shown in Figure 1 as lines of different colors, passing through Baofeng wharf of Yangjiang, Daya Bay station, No.8 wharf of Qingdao port Taiyuan station, and then Jiuquan station, meeting the requirements of multiple objectives.

This section is not mandatory but can be added to the manuscript if the discussion is unusually long or complex.

#### 5.1.1. Determination of Total Objective Function by Analytic Hierarchy Process

In planning single transportation, this step mainly lies in allocating resources, personnel, and equipment for each road section. Considering risk, cost, efficiency, other factors, and various resource constraints, we establish objective functions and constraints and carry out resource allocation and time planning for different sections. For the multi-mode transportation process of determining the transportation volume, the number of transportation tools and transportation personnel required under the fixed transportation mode is determined based on the full load of transportation tools. There is no room for optimizing transportation personnel cost, transportation cost, and transportation time. Therefore, in this case, the optimization items are various loading and unloading resources (such as loading and unloading personnel, loading and unloading equipment) in the allocation of each transfer site. If the numbers of loading and unloading equipment at five transfer sites are 1 to 5, the numbers of loading and unloading personnel at five transfer points are 1 to 5.

The single transportation plan needs to determine the risk, time, and cost models. The risk model mainly describes the probability of danger in the transportation process and the consequences after the threat. The measurement indicators are the probability of an accident in the path, population density, road impedance, and emergency rescue.

Transportation time is also an essential factor in the transportation process. Transportation time comprises loading and unloading time, transportation time, and typical scenes delay time. The delay time mainly includes sea ice, fishing season, ocean current, and typhoon, which may be encountered in maritime transportation and large-scale activities and road collapse faced by road transportation.

Transportation cost is an essential condition that needs to be controlled in the transportation process, mainly including personnel, container, equipment, and transportation tool costs. Single transportation planning needs to comprehensively consider the above four costs for simple summation to minimize the total cost for cost estimation and control.

The transportation risk, time, and transportation cost are weighted as the penalty items of the objective function. After the risk, time, and cost preferences are determined by the expert scoring method, the weight value is determined by the analytic hierarchy process. The resource allocation plan that minimizes the objective function is the initial transportation plan. The above three items are not only put into the objective function but also constrained by constraints to ensure the safety and feasibility of transportation.

The parameters required during function setting are as follows (see Table 1):

(1) Transport risk function (R).

During the transportation of goods, if an accident occurs, it will not only cause particular harm to the surrounding environment and personnel but also can cause irreversible damage to various buildings. Therefore, the risk can be measured according to the four indicators of accident probability, population density, road impedance, and emergency rescue capacity. Finally, the total risk can be calculated and compared according to the risk sum of each road section. For the road section risk in the path network, the value of the evaluation index can be determined by the scoring method. Then, the weight coefficient of each evaluation index can be given by the analytic hierarchy process. The specific process is as follows:

① Use a pre-decided relative weight scale to construct judgment matrix A:
  (20) $$A=\left[\begin{array}{cccc} 1 & 5 & 3 & 5\\ 1/5 & 1 & 1/3 & 1/3\\ 1/3 & 3 & 1 & 1/3\\ 1/5 & 1/3 & 3 & 1 \end{array}\right]$$
② Hierarchical single sorting to determine the weight vector. The maximum eigenvalue of the matrix and its corresponding eigenvector can be obtained as:
  λ = 4.1154(21)
  (22) $$\vec{l}=(0.58,0.08,0.15,0.19)$$
③ Consistency test.
  (23) $$\mathrm{CI}=\frac{\lambda -n}{n-1}=0.0385$$
  (24) $$\mathrm{CR}=\frac{\mathrm{CI}}{\mathrm{RI}}=0.0428$$

As CR < 0.1, it can be determined that the matrix has complete consistency through a consistency test, and the eigenvector is taken as the weight vector. Therefore, the risk determined by the analytic hierarchy process is:

R = 0.58r1 + 0.08r2 + 0.15r3 + 0.19r4(25)

In the formula, ri is the scoring value of the ith influencing factor.

(2) Transport time function (C).

Total time spent = Loading and unloading time + Transportation time(26)

Transportation time = Calculated transportation time + Delay time of typical scenario

Among them, the transportation distance and average transportation time are calculated as 65 days for the transportation time. As for the loading and unloading time, adding ten loading and unloading personnel can reduce the loading and unloading time by 2%, while adding a set of loading and unloading equipment can reduce the loading and unloading time by 5%. We take the median value of the traveling speed in the path to calculate the transportation time. The road transportation takes 25 days; the seaway transportation takes 20 days, the railway transportation takes 15 days, and the total transportation time is 60 days. For the delay time of typical scenarios, half of the sea transportation will encounter a freezing season during the whole transportation process. According to the plan, the sea transportation time will be increased by 2 days. According to the plan, if the entire journey experiences a fishing season, the seaway transportation time will be increased by 2 days. If there is a typhoon during the transportation, it will need to stop for 1 day each time.

In this case, the basic time for five sets of loading and unloading at the five transfer sites is 10 days. The basic equipment for loading and unloading is 5 sets, and the basic personnel for five sets of loading and unloading is 50 people.

Therefore, the transport time function is:

T = 10 × [1− (C1 + C2 + C3 + C4 + C5 − 50) × 0.2% − (A1 + A2 + A3 + A4 + A5 − 5) × 5%] + 65(27)

(3) Transportation cost function (C).

Total transportation cost = personnel cost + container cost + equipment cost + transportation tools cost

personnel cost = transportation personnel cost + loading and unloading personnel cost

(4) Total objective function.

The weights of transportation cost, transportation risk, and transportation time are 0.222, 0.667, and 0.111, respectively. Therefore, the total objective function is:

Min Z = 0.222 × C + 0.667 × R + 0.111 × T(28)

In the formula, C, T, and R represent Equations (25)–(27), respectively.

(5) Constraints.

According to the actual situation, the maximum number of loading and unloading equipment is 70 sets and the maximum number of loading and unloading personnel is 1500, that is:

A1 + A2 + A3 + A4 + A5 ≤ 70(29)

C1 + C2 + C3 + C4 + C5 ≤ 1500(30)

#### 5.1.2. Gradient Descent Method for Solving Transportation Plan

**Figure 5:**
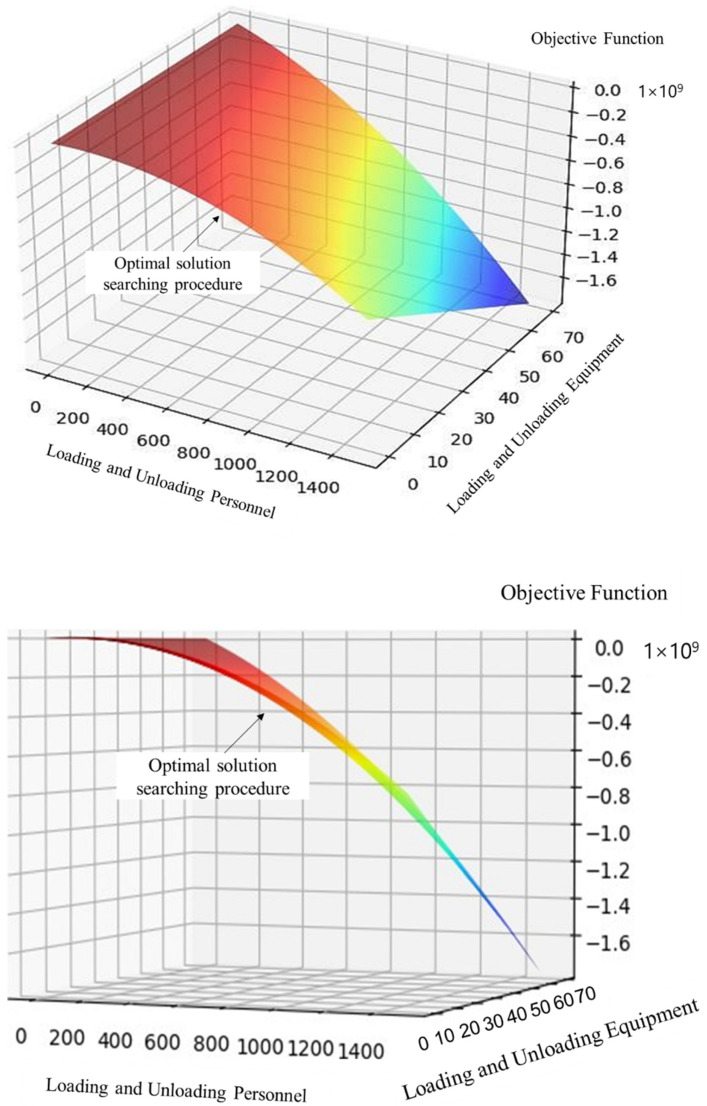
Objective function image from the gradient descent method.

Fuzzy eclectic planning is one of the previously widely used shortest path algorithms. It aims to blur the boundaries of linear constraints so that people can find optimized conditions and extremums under more relaxed conditions. This paper uses the gradient descent algorithm to solve the large-scale transportation optimization problem and search for the optimal solutions (see Figure 5). In fact, the advantages of gradient descent are even more pronounced if the data size is more extensive, which is why gradient descent is crucial for problems relying on large datasets.

When the objective function reaches the lowest point, the transportation time is 70 days. The total number of loading and unloading personnel required at the five transfer sites is 1280, and the total number of transportation personnel is 120. The total cost is 7.702 million, and the total number of loading and unloading equipment is 70 sets. We also calculate the results with the fuzzy eclectic planning algorithm for comparison purposes. The results are summarized in Table 2. It can be found that the gradient descent algorithm applied in our study outperforms the fuzzy eclectic planning for the shortest path problem. More specifically, the gradient decent algorithm improves the total transportation time by 7.89%, the entire personnel by 10.35% to 17.31%, total cost by 13.58%, and total equipment by 7.89%. The results demonstrate the advantages of the gradient descent algorithm for large-scale network optimizations.

According to the principle of equal distribution of resources at each transfer site, the transportation plan and layout plan of this case are as follows (Table 3 and Table 4):

The method addresses the transport of dangerous goods under multi-modal transport conditions, with the transport process covering three modes of transport: sea, road, and rail. The method calculates the most suitable transport solution based on three influencing factors: transport cost, transport risk, and transport time, and it is designed to help transport decision makers to develop transport solutions that include the generation of transport routes and the deployment of transport resources, as well as individual transport solutions that include schedules, layouts, and transport overview maps.

## 6. Conclusions

This paper analyzes and optimizes the transportation mode and path decision-making plan in the multi-mode transportation network. Based on the structure and attributes of the network, the network graph of different transportation modes is integrated through transit links to build a multi-mode network system. The transportation process covers three transportation modes: seaway transportation, highway transportation, and railway transportation. On this basis, the optimal path is selected by using the Dijkstra algorithm and the multi-objective optimization algorithm. Among them, the indexes adopted by multi-objective optimization include transportation cost, time, and risk. Through the deep analysis of the influencing factors of plan formulation, including the transportation cost function, time function, and risk function, the multi-objective function is transformed into a single objective function by the analytic hierarchy process. The gradient descent method is used to solve the plan. The overall transportation plan is formulated, including transportation path generation and resource allocation.

In our future studies, we will expand the transportation network by considering more modes such as air transportation during the modeling. We will also develop more efficient and effective solutions for optimizing the joint decision of transportation path and mode. Furthermore, we will compare different optimization methodologies to find the better one for our modeling purpose. The method currently used in the paper does not consider the effects of uncertainties such as time uncertainty and risk uncertainty when constructing a multi-objective decision model, and further research is needed on the transport mode and route decision options for multi-modal transport networks under uncertain environmental factors. We believe that future studies can focus on those issues.

## Figures and Tables

**Table 1:** Key parameters for model settings.

Basic Item	Basic Parameter
Total amount of single freight	10 t
Unit container capacity	1 t
Cost of special container	1 million/a
Transportation/loading and unloading personnel wages	1000 yuan/day
Highway and seaway replacement equipment	50,000/set
Highway and railway replacement equipment	40,000/set
Highway loading/unloading equipment	20,000/set
The speed of large container ships on the sea	36~52 km/h
The speed of freight cars on the highway	60~100 km/h
The speed of freight trains	70~100 km/h
Unit cost of highway transportation	0.6 t/km
Unit cost of seaway transportation	0.08 t/km
Unit cost of railway transportation	0.15 t/km
Highway transportation mileage 1	415 km
Highway transportation mileage 2	845 km
seaway transportation mileage	2263 km
Railway transportation mileage	1491 km

**Table 2:** Result of the transportation plan from different models.

Result Item		Gradient Descent	Fuzzy Eclectic Planning	Improvement
time		70 day	76 day	7.89%
Personnel	Loading and unloading personnel	1280 people	1548 people	17.31%
	Transportation personnel	120 people	134 people	10.45%
Cost		7.702 million	8.912 million	13.58%
Loading and unloading equipment		70 sets	76 sets	7.89%

**Table 3:** Procedure of the transportation plan.

Result Item		Specific Item	Specific Data
time		Daya Bay nuclear power plant (starting point loading)	1 day
		Daya Bay nuclear power plant—Baofeng Wharf of Yangjiang (road transportation)	6 day
		Baofeng Wharf of Yangjiang (conversion between highway and seaway transportation)	1 day
		Baofeng Wharf of Yangjiang—No.8 wharf of Qingdao port (seaway transportation)	25 day
		No.8 wharf of Qingdao port (conversion between highway and seaway transportation)	1 day
		No.8 wharf of Qingdao port—Taiyuan station (highway transportation)	19 day
		Taiyuan station (conversion between highway and railway transportation)	1 day
		Taiyuan station—Jiuquan station (railway transportation)	15 day
		Jiuquan station (terminal unloading)	1 day
personnel	Loading and unloading personnel	Daya Bay nuclear power plant (starting point loading)	256 people
		Baofeng Wharf of Yangjiang (conversion between highway and seaway transportation)	256 people
		No.8 wharf of Qingdao port (conversion between highway and seaway transportation)	256 people
		Taiyuan station (conversion between highway and railway transportation)	256 people
		Jiuquan station (terminal unloading)	256 people
	Transportation personnel	Daya Bay nuclear power plant—Baofeng Wharf of Yangjiang (road transportation)	10 people
		Baofeng Wharf of Yangjiang—No.8 wharf of Qingdao port (seaway transportation)	50 people
		No.8 wharf of Qingdao port—Taiyuan station (highway transportation)	10 people
		Taiyuan station—Jiuquan station (railway transportation)	50people
cost		Personnel cost	3.53 million
		Container cost	2 million
		Equipment cost	2.16 million
		Transportation cost	12,000
Transportation tool		Truck	10 vehicles
		Vessel	1 ship
		Train	10 sections
Loading and unloading equipment		Daya Bay nuclear power plant (starting point loading)	12 sets
		Baofeng Wharf of Yangjiang (conversion between highway and seaway transportation)	12 sets
		No.8 wharf of Qingdao port (conversion between highway and seaway transportation)	12 sets
		Taiyuan station (conversion between highway and railway transportation)	12 sets
		Jiuquan station (terminal unloading)	12 sets

**Table 4:** Solution of the transportation schedule.

Time	Transportation Matters	Loading and Unloading Equipment	Loading and Unloading Personnel	Transportation Personnel	Transportation Tool
1.2–1.3	Daya Bay nuclear power plant (starting point loading)	12 sets	256 people		
1.3–1.9	Daya Bay nuclear power plant—Baofeng Wharf of Yangjiang (road transportation)			10 people	5 vehicles
1.9–1.10	Baofeng Wharf of Yangjiang (conversion between highway and seaway transportation)	12 sets	256 people		
1.10–2.5	Baofeng Wharf of Yangjiang—No.8 wharf of Qingdao port (seaway transportation)			50 people	1 ship
2.5–2.6	No.8 wharf of Qingdao port (conversion between highway and seaway transportation)	12 sets	256 people		
2.6–2.25	No.8 wharf of Qingdao port—Taiyuan station (highway transportation)			10 people	5 vehicles
2.25–2.26	Taiyuan station (conversion between highway and railway transportation)	12 sets	256 people		
2.26–3.13	Taiyuan station—Jiuquan station (railway transportation)			50 people	10 sections
3.13–3.14	Jiuquan station (terminal unloading)	12 sets	256 people		

## Data Availability

The study did not report any data.
